# Supplementary material for: Deletion of wfs1 Impairs Oligodendrocyte Precursor Cells Dorsal Distribution and Myelination Through the wfs1-hmgcs1 Axis in Zebrafish
Source: Biology (Basel). 2026 Aug 16;15(16):1402. doi: 10.3390/biology15161402 (PMC13510151; doi:10.3390/biology15161402)
Supplement: Supplementary file 1 [file biology-15-01402-s001.zip › File S1-Transcriptome sequencing data and differentially expressed genes (DEGs) in wfs1-- zebrafish at 5 dpf/2.KEGG/wfs1KOvsWT/UP/src/dre04916.html]

dre04916


- dre:192322

- Up regulated genes

336121(1.42904963424916)

- dre:323509

- Up regulated genes

323509\_1(2.61236898874666)

- dre:553181

- Up regulated genes

553181\_1(2.90602507971719)

- dre:58072

- Up regulated genes

58072\_1(2.20804115837263)

- dre:566841

- Up regulated genes

566841(1.91411798034227)

- dre:100006675

- Up regulated genes

100006675(1.63301233543285)

- dre:100538072

- Up regulated genes

100538072(1.10439883897206)

- dre:568726

- Up regulated genes

568726(1.14419230813088)
- dre:560807

- Up regulated genes

560807(1.71247301071773)

- dre:100006675

- Up regulated genes

100006675(1.63301233543285)

- dre:556087

- Up regulated genes

556087(1.25789886083115)

Close
